# J-SPACE: a Julia package for the simulation of spatial models of cancer evolution and of sequencing experiments

**DOI:** 10.1186/s12859-022-04779-8

**Published:** 2022-07-08

**Authors:** Fabrizio Angaroni, Alessandro Guidi, Gianluca Ascolani, Alberto d’Onofrio, Marco Antoniotti, Alex Graudenzi

**Affiliations:** 1 Dept. of Informatics, Systems and Communication, Univ. of Milan-Bicocca, Milan, Italy; 2 Department of Mathematics and Geosciences, Univ. of Trieste, Trieste, Italy; 3 Bicocca Bioinformatics, Biostatistics and Bioimaging Centre (B4), Milan, Italy; 4 Inst. of Molecular Bioimaging and Physiology, National Research Council (IBFM-CNR), Segrate, Italy

**Keywords:** Cancer Evolution, Stochastic Simulation, Spatial dynamics, Next-generation sequencing

## Abstract

**Background:**

The combined effects of biological variability and measurement-related errors on cancer sequencing data remain largely unexplored. However, the spatio-temporal simulation of multi-cellular systems provides a powerful instrument to address this issue. In particular, efficient algorithmic frameworks are needed to overcome the harsh trade-off between scalability and expressivity, so to allow one to simulate both realistic cancer evolution scenarios and the related sequencing experiments, which can then be used to benchmark downstream bioinformatics methods.

**Result:**

We introduce a Julia package for SPAtial Cancer Evolution (J-SPACE), which allows one to model and simulate a broad set of experimental scenarios, phenomenological rules and sequencing settings.Specifically, J-SPACE simulates the spatial dynamics of cells as a continuous-time multi-type birth-death stochastic process on a arbitrary graph, employing different rules of interaction and an optimised Gillespie algorithm. The evolutionary dynamics of genomic alterations (single-nucleotide variants and indels) is simulated either under the Infinite Sites Assumption or several different substitution models, including one based on mutational signatures. After mimicking the spatial sampling of tumour cells, J-SPACE returns the related phylogenetic model, and allows one to generate synthetic reads from several Next-Generation Sequencing (NGS) platforms, via the ART read simulator. The results are finally returned in standard FASTA, FASTQ, SAM, ALN and Newick file formats.

**Conclusion:**

J-SPACE is designed to efficiently simulate the heterogeneous behaviour of a large number of cancer cells and produces a rich set of outputs. Our framework is useful to investigate the emergent spatial dynamics of cancer subpopulations, as well as to assess the impact of incomplete sampling and of experiment-specific errors. Importantly, the output of J-SPACE is designed to allow the performance assessment of downstream bioinformatics pipelines processing NGS data. J-SPACE is freely available at: https://github.com/BIMIB-DISCo/J-Space.jl.

## Background

Cancer development is an evolutionary process characterised by the emergence, competition and selection of cell subpopulations exhibiting certain functional advantages with respect to normal cells (i.e., cancer clones). Each subpopulation originates from specific somatic alterations of the (epi)genome, which are typically referred to as *drivers* [1]. Drivers confer cancer cells an increased *fitness*, for instance in terms of enhanced replication rate, ability to evade the immune system, avoid apoptotic signals, or ability to diffuse, as well as resistance to therapeutic interventions [2].

Both cancer and normal cell subpopulations compete in a complex interplay occurring within the micro-environment and are continuously either selected or purified in Darwinian evolution scenario, hence resulting in the high levels of *intra-tumour heterogeneity* that are observed in most cancer types [3]. In addition, during replications, both normal and cancer cells acquire and accumulate a large number of neutral mutations, named *passengers*, which do not alter their overall fitness. In principle, all mutations can be used as *barcodes* to track the clonal composition and evolution in time, by performing variant calling from DNA- and RNA- Next-Generation Sequencing (NGS) experiments generated from tissue biopsies or from patient-derived cell cultures, xenografts or organoids, and this can be done either at bulk or single-cell resolution [4].

In recent years, many computational methods have been developed to exploit the increasing amount of NGS data, either to detect point mutations, indels, copy number variations and structural variations [5–7], perform clonal deconvolution [8, 9] or return evolutionary models [10–14].

However, despite the impressive number of works exploiting NGS data, the effects of the combination of the experimental protocols many parameters with those of the bioinformatics pipelines remains unexplored and may lead to biases that affect any downstream analysis [15]. Therefore, developing a standardised procedure to assess such biases and validate the results is necessary, and simulations are one of the most effective tools available to achieve these goals [16]. For this reason, a significant number of software tools have been recently developed and released to simulate either (*i*) the molecular (genomic) evolution of tumours or (*ii*) the (spatial) population dynamics of multi-cellular systems.

Many approaches simulate the *genomic evolutionary dynamics* of tumours, typically by considering branching processes (or coalescent models) that underlie the origination and accumulation of Single-Nucleotide Variants (SNVs) and other genomic alterations [17–21]. This is often achieved by relying on the Infinite Sites Assumption (ISA) [22, 23], which however presents some important limitations. First, it is known that the ISA might be violated and that such violations are relatively common in several cancer types, for instance due to convergent evolution and back mutations [24, 25]. Second, distinct processes underlie the nucleotide substitution patterns that are observed in most cancer types, also known as mutational signatures [26, 27]. Such processes can be endogenous (e.g., APOBEC deaminase activity causes mainly C to T substitutions) or exogenous (e.g., tobacco smoke causes mainly C to A substitutions), and their activity may change during the development of the disease [28]. These processes cannot be realistically simulated without a finite-sites model, where sites are not independent. Finally, large structural variations such as gene fusions and copy number alterations, which are essential for clonal/lineage tracking [29–31] cannot be represented using the ISA. Importantly, most frameworks modelling the genomic evolution of tumours do not explicitly consider the spatial dynamics of cancer cells, which is known to have a dramatic impact on the overall evolution of tumours and on the related samplings [32, 33].

A different class of approaches comprises several simulation tools that have been developed to represent the *spatial population dynamics* of cells and tissues and the microscopic interaction among cells, via plausible biophysical representations [34]. For instance, agent-based models [35], cellular automata [33, 36, 37], finite elements simulations [38, 39], and hybrid approaches [40] have been used to investigate the influence of spatial constraints on cancer development. Other simulation frameworks focus on the mechanical interactions among neighbours cells [41], the interaction between different cell (sub)types, e.g., between cancer cells and the stroma [42, 43], the metabolic interplay [44–46], or the specialisation/differentiation processes [47–49].

Notably, some recent attempts combine the simulation of genomic evolution with that of spatial dynamics of tumours, yet they rely on the ISA to produce their results [33].

In this extremely lively field, we observe a shortage of efficient spatial cancer simulation tools capable to generate a broad spectrum of in-silico scenarios, while producing a rich set of standardised outputs usable in downstream bioinformatics pipelines. In principle, such tool should be able to simulate a large number of cells and realistic sequencing experiment scenarios, and abide distinct spatial constraints, microscopic interactions and substitution models. To fill this gap, we introduce the SPAtial Cancer Evolution SIMulator (J-SPACE), a Julia package that exploits optimised algorithms for the simulation of spatio-temporal evolution of tumours, spatial sampling of cells, molecular evolution of sequences under different substitution models, with the possibility to include indels. By relying on the NGS read simulator ART [50], J-SPACE generates synthetic reads in standard formats such as FASTA, ALN, SAM and FASTQ, giving the possibility of a straightforward implementation of bioinformatics benchmarking pipelines.

## Implementation

**Fig. 1:**
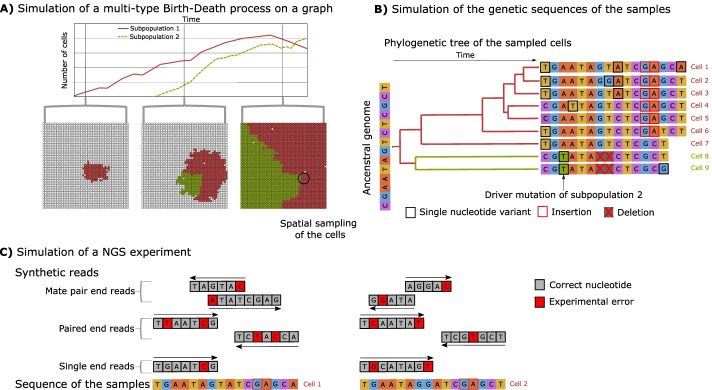
The J-SPACE framework. Schematic representation of J-SPACE. **A** First, the algorithm simulates the spatial growth of the cells over an arbitrary graph. Then, J-SPACE simulates a spatial sampling (black circle) at a given time point. **B** J-SPACE reconstructs the phylogeny of the sampled cells (i.e., the leaves of the tree) and, given an ancestral genome, it generates the ground-truth sequence of the sampled cells using various substitution models. **C** A NGS experiment is simulated to return synthetic reads as outputs

A schematic workflow of J-SPACE is depicted in Fig. 1. J-SPACE relies on an Optimized Gillespie Algorithm (OGA) to simulate the spatial dynamics of cells populations [51]. The dynamics of the spatio-temporal evolution of a tumour is modelled by a stochastic continuous-time multi-type Birth-Death (BD) process over an arbitrary graph.

J-SPACE can work with a 2D or 3D regular lattice, but it can also work with any arbitrary graph (which, of course, must be approprately interpreted). In a simulation, all cells can acquire and accumulate random mutations over time; rarely, some of these mutations enhance the birth rate (i.e., the fitness) of all descendants. These mutations are the so-called ”drivers”. Then J-SPACE mimics the sampling of a portion of cells (e.g., a biopsy) and after computing the phylogenetic tree of such cells, it simulates the evolution of nucleotide sequences along the phylogeny, in order to obtain the genetic sequences of all sampled cells [52–54]. To model the mutation evolutionary dynamics, J-SPACE allows the user to employ any of the following.

- An Infinite Sites model [55].
- A set of finite-sites models (JC69 [56], F81 [57], K80 [58], HKY85 [59], TN93 [60], K81 [61])
- A custom time-dependent trinucleotide substitution model using a linear combination of mutational signatures from the COSMIC database [26].

In addition, J-SPACE allows to simulate indels in any of the finite-sites models.

Finally, J-SPACE mimics NGS experiments by calling ART [50] to generate sequencing reads. The user can run any configuration of ART: it is possible to simulate single-end, paired-end/mate-pair reads, with various error models and different values of coverage for different sequencing platforms.

J-SPACE provides the following outputs:

- The state of the lattice/graph at any time of the simulation.
- The Ground Truth (GT) sequence of the sampled cells as FASTA files.
- The list of mutations for each sampled cell.
- The GT phylogenetic tree of the sampled cells in Newick format.
- The mutational tree of the driver mutations (if present), where the nodes represent mutations and edges model the accumulation temporal direction as proposed in [11, 13].
- The simulated NGS reads as FASTQ files.
- The alignment file, which maps the noisy reads on the sequences of the sampled cells both in formats SAM and ALN.
- The GT alignment file, with the reads without noise in SAM format.

All parameters of J-SPACE are managed by means of two simple input textual files, the first one used to set up general configuration parameters (e.g., file paths, plotting and output options, etc.), the second one including all simulation parameters. For a complete description of the parameters and usage examples, please refer to https://github.com/BIMIB-DISCo/J-Space.jl.

### Generating spatial cancer dynamics

In J-SPACE, the spatio-temporal dynamics of a multi-cellular system is modeled as a stochastic process over an arbitrary graph embedded in ${\mathbb {R}}^D$, in which each node can be empty or occupied by a single cell. More in detail, the graph is composed by a set of points in ${\mathbb {R}}^D$. A pair of points can interact if their distance in ${\mathbb {R}}^D$ is smaller than a positive real number *J*, called the range of interaction. By connecting each point with the points within distance less than *J*, we obtain a graph that represents the finite elements space where the dynamics occurs.

Each point has an associated state: an integer in $\{0,1,\dots n_\text {pop}\}$, where 0 indicates an empty node, and $i=1,\dots ,n_\text {pop}$ indicates that a node is occupied by the $i^\text {th}$ subpopulation present in the system. Subpopulations here represent the cells bearing the same set of driver mutations (see below), i.e., cancer clones, Accordingly, all cells belonging to the same subpopulation will have the same state. Note that, by design, subpopulation $i=1$ does not harbour any driver mutation, so it can be considered either as the wild type (e.g., healthy cells) or as the ancestral cancer subpopulation.

As in a standard BD model, two probabilistic moves are possible.

1. *Death*, that is a constant stochastic process where sites become vacant (state $=0$) at a constant rate $\beta$ per unit of time.
2. *Birth*, that represents an *interaction* between two nodes of the lattice.

In J-SPACE the birth event is modeled as follows: a parent cell divides into two daughter cells with a rate equal to $\alpha$ per unit of time, occupying the location of the parent cell and that of randomly chosen position among its nearest neighbours node. When studying the cells’ spatial interaction, it is crucial to simulate processes such as the replication inhibition due to the absence of space, e.g., the exclusion process [41, 62]. For this reason, J-SPACE implements three different kinds of interaction rules.

- The contact process [63].
- The voter model on heterogeneous graphs [64].
- The hierarchical voter model.

In the contact process, a cell can duplicate itself only if it has an empty node in its neighbourhood: in this scenario, there is a strong replication inhibition due to spatial constraints, while the advantage of driver mutations is softened. In the voter model, the exclusion principle is dropped: a cell can “kill” one of its neighbours and substitute it with one of its daughters: this situation is equivalent to a Moran process, and it helps generating highly correlated spatial clusters [65]. Finally, the hierarchical voter model is akin to the previous one, but a cell can “kill” and replace one of its neighbours only if it has a greater birth rate (e.g., it bears more driver mutations). This situation represents a tissue where the growth of wild-type cells is inhibited by its neighbourhood, while cells bearing driver mutations are unregulated and can proliferate even if their neighbourhood is full.

J-SPACE simulates the emergence and accumulation of driver mutations, which also allow us to define the subpopulations (i.e., clones) interacting within the system. We set a probability $\mu _{\text {dri}}$ that one of the two daughter cells acquires a new driver mutation. Each newly acquired driver mutation provides the cell with a birth rate increase and, in particular, we suppose that such increase is distributed as a (positively truncated) Gaussian variable with both mean and standard deviation provided as input. Since we here assume that cells inherit the same mutations of their parental cell, every distinct subpopulation will have a different birth rate $\alpha _i$ per unit of time, which is equal to the linear combination (all weights $=1$) of the birth rate of the wild type and the birth rate advantages of the driver mutations of the specific subpopulation. In addition, in order to give the user the possibility to control the evolution of cancer subpopulations, it is possible to provide the mutational tree of the drivers [11, 13] and the birth rate of each subpopulation as inputs to J-SPACE. Note that, in this case, the simulation can lead to the emergence of subtrees of the input mutational tree, due to the stochastic dynamics of the framework.

Many theoretical approaches that optimise an event based simulation of a BD process on a graph [66] have been developed in the past. Despite the outstanding results of these methods, minimal deviations from statistically exact prescriptions can lead to uncontrolled biases [51, 66, 67], and Montecarlo simulations are the only statistical methods to integrate these system in every configuration [66].

**Fig. 2:**
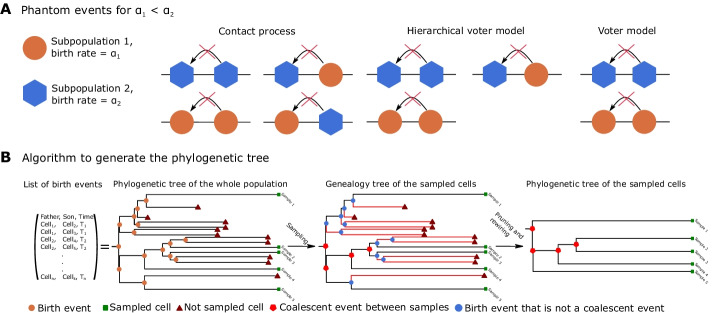
Phantom events and the reconstruction of phylogenetic trees. **A** Pictorial representation of the possible phantom events in a simulation with two different subpopulations. **B** Simplified scheme of the algorithm that generates the ground-truth phylogenetic tree from the list of birth events. First, the algorithm prunes the branches the leaves of which are not sampled (in red), then it removes the remaining edges that are not coalescent events

A straightforward implementation of an event based simulation (i.e., the Doob-Gillespie algorithm [68, 69]) in networks including a large number of nodes, quickly becomes computationally cumbersome. For this reason, J-SPACE relies on an OGA that is borrowed from methods originally developed for the simulation of Markovian epidemic processes on large networks [51]. Briefly, an OGA introduces *phantom events* that are those events that violate the chosen interaction rule. The algorithm follows the standard procedure of an event-based simulation on a graph, but it evaluates the total rate of events considering both phantom and non-phantom events. It randomly picks the waiting time of the next event from a exponential distribution. An event is chosen with a probability proportional to its rate, if such event is a phantom event only the time is updated, otherwise both the time and the state of the system are updated. Phantom events are differently defined for every interaction rule included in the implementation of J-SPACE. For the contact process, a phantom event occurs when a cell replicates itself occupying a non-empty node; for the voter model when a cell replicates itself occupying a node that is inhabited by a cell of the same subpopulation; for the hierarchical voter model when a cell replicates itself occupying a node that is occupied by a cell with equal or higher birth rate (see Fig. 2A for an example).

The algorithm then follows the usual procedure of an event-based simulation. It evaluates the total rate of events considering both phantom and non-phantom events. It randomly pick the waiting time of the next event from a exponential distribution. An event is chosen, if such event is a phantom event only the time is updated, otherwise both the time and the state are updated. The main computational improvement of OGA with respect to the standard the Doob-Gillespie algorithm is that the set of the nodes that could be occupied by a cell is not evaluated every time an event occurs. By introducing such phantom events, the computational time may improve by several orders of magnitude with respect to the standard implementation. Moreover the difference increases with the number of nodes of the graph [51].

Importantly, J-SPACE introduces the possibility of performing an arbitrary number of bottleneck events, in which a user-defined portion of the tumour is wiped-out. This can be achieved by specifying the time and the size of such events (i.e., the proportion of the population that will survive to these events). This simulation option allows one to mimic the impact of simple pharmacological interventions, and sets the basis for future developments involving more realistic simulations based on pharmacokinetic and pharmacodynamic models [70].

Finally, J-SPACE returns the subpopulation dynamics (in a textual format) and the configuration of the graph at any time as output.

### Generating phylogenetic trees

After the simulation of the spatial dynamics, J-SPACE offers the possibility of sampling a user-selected number of randomly distributed cells or a circular/spherical region (2D/3D scenario) with a user-selected radius, in order to simulate a biopsy and obtain the list ${\mathcal {S}}$ of sampled cells.

J-SPACE reconstructs the phylogenetic tree of the sampled cells by computing their *genealogy tree*, i.e., a graph ${\mathcal {G}} = (V, E)$. In ${\mathcal {G}}$ the set of the nodes *V* is composed by the nodes of degree 1 (i.e., the sampled cells ${\mathcal {S}}$ and their least recent common ancestor) and by the nodes of degree 2 or 3 that are ancestors of the sampled cells. The set of edges *E* represents the parental relations between cells. To reconstruct ${\mathcal {G}} = (V, E)$, J-SPACE saves the following lists while computing the spatial dynamics.

- ${{\mathcal {P}}}{{\mathcal {A}}}_m$, i.e., the label of the parental cell in the $m^{\text {th}}$ birth event.
- ${{\mathcal {D}}}{{\mathcal {A}}}_m$ the list of the labels of the two nodes occupied in the $m^{\text {th}}$ event.
- ${\mathcal {T}}_m$ the timestamp of the $m^{\text {th}}$ event.

Note that the label associated with a cell is unique during the simulation and we assume that, when dividing, a cell dies and generates two cells with two distinct new labels. Since one parent cell always generates two daughter cells, this list defines a binary phylogenetic tree where the leaves are either dead cells or cells present at the time of sampling.

Then, J-SPACE scrolls backwards the lists ${{\mathcal {P}}}{{\mathcal {A}}}_m$ and ${{\mathcal {D}}}{{\mathcal {A}}}_m$, and it obtains ${\mathcal {G}} = (V,E)$ by registering the non-phantom birth and mutation events that are in the past of the sampled cells (see Fig. 2B).

Since the nodes of ${\mathcal {G}}$ with degree 3 are the internal nodes of a phylogenetic tree (i.e., the birth or mutation events that are coalescent events between the samples), whereas the nodes with degree 1 are either the root or the leaves of such tree, by deleting all the nodes with degree equal to 2 (i.e., the birth or mutation events that are not coalescent events between the sampled cells) and redrawing the edges between the remaining node coherently, J-SPACE obtains the ground truth phylogenetic tree of the sampled cells ${\mathcal {S}}$ (see Fig. 2B). Finally, the GT phylogenetic tree is returned in Newick format.

### Genotype of sampled cells

As specified in the Background Section, the large majority of mutations that can hit a given cell during its lifetime have no functional effect (i.e., they are passengers), and only a very small number of events implies a phenotypic change. From the computational perspective, it would be inefficient to explicitly simulate the evolution of nucleotide sequences during the computation of the spatial dynamics of the subpopulations. There are two reasons for this: *i)* a large number of cells implies an huge number of nucleotides, and therefore a huge computational load to compute all the genetic events, and *ii)* simulate the sequence of non-sampled cells would be a waste of computational resources.

For these reasons, J-SPACE simulates *a posteriori* the evolution of nucleotide sequences along the phylogeny of the sampled cells [52–54]. Assuming that mutations are independent among sites, and that the mutational process could be modelled as a continuous-time Markov chain, J-SPACE simulates the mutational events via the exact Doob-Gillespie algorithm, both infinite and finite-sites models are implemented. In the case of a finite-sites model, also indels could be simulated. Note that using finite-sites models allow for simulating back-mutations and multiple mutations at a site, although this comes at the cost of decreased computational performance [53].

To simulate the molecular evolution, J-SPACE uses the phylogenetic tree of the sampled cells ${\mathcal {S}}$ and an ancestral genome, which can be given by the user or generated randomly, given the length of the genome *L* and the frequencies of the nucleotides (e.g., $\nu _A =\text {number of nucleotides ``A''}/L$) . In the case of the infinite-sites model, J-SPACE generates the number of mutations for each branch of the phylogenetic tree in the following way: starting from time equal to zero, the time of the next event is picked randomly from an exponential distribution with a rate equal to the product of the length of the sequence and the neutral mutational rate ($\mu _\text {neut}$). Then the time is updated. When the elapsed time is longer than the branch length, the number of events is the number of mutations associated with such a branch. The genotype of a sample is retrieved by enumerating the edges of the paths between the given ancestral genome and the sample itself, and associating to it all the mutations present on the edges of the path. Note that in this case each branch is considered independent and each mutation is considered unique, for this reasons back-mutations or multiple hits are not possible. This approximation is useful to have fast simulations where the genome is very long, the mutational rate is very low, and the total simulated time is long.

In the case of finite-sites models, J-SPACE takes as input the matrix of instantaneous rates for different substitution models and, for each branch of the phylogenetic tree, the evolution of the genome is evaluated. Given a branch between two nodes, we start from the sequence of the parent cell and set the time *t* equal to 0. Then, we evaluate the total substitution rate for the entire sequence as the sum of the rate of all possible events, i.e.:

1 $$\begin{aligned} R = \sum _{k = 1}^L\left( \mu _\text {indel}+ \sum _{i \in \{A,T,C,G\} \ne s(k)} q_{s(k),i}\right) , \end{aligned}$$

where *L* is the length of the sequence, *s*(*k*) is the state on the sequence at position *k*, $q_{ s(k),i}$ is the rate of substitution from the azotate base *s*(*k*) to the base *i* per unit of time, and $\mu _\text {indel}$ is the indel rate per site per unit of time. Subsequently, the time $\tau$ of the next event is picked randomly from an exponential distribution with rate *R*, and the type of event is randomly chosen with a probability proportional to its total rate. For example, the probability that a substitution C>T is chosen is $P_{C>T} =\sum _{k = 1}^L q_{C,T} /R$. After that, the time is updated to $t = t + \tau$ and the rate and the sequence are updated. The simulation is continued till the elapsed time *t* is longer than the branch length. This procedure is performed on each branch of the phylogenetic tree starting from to the root and moving toward the leaves (i.e., the samples).

In the case the event is an indel, following [54] we suppose that its length has a size distributed as a Lavalette law, where the probability of having an indel of length *l* is proportional to $[l L_\text {indel}/ (L_\text {indel}- l + 1)]^ {-a}$. In this case the user should give the maximum possible length of an indel $L_\text {indel}$ and the parameter *a* of the Lavalette distribution. Since this exact simulation is very time consuming, and possible only for small trees, it is possible to simulate the substitutions and the indels as independent processes [53, 54]. In this case J-SPACE compute the SNVs with a substitution model, and afterwards the indels are generated along phylogenetic tree branches as before.

J-SPACE implements the following substitution models: JC69 [56], F81 [57], K80 [58], HKY85 [59], TN93 [60], and K81 [61].

To simulate the SNVs, it is also possible to generate a time-dependent trinucleotide substitution model starting from the Single Base Substitution (SBS) signatures present in the COSMIC database [71]. In this case, the user should specify the of list of desired signatures (i.e., their label in the COSMIC database $S_1,\dots ,S_n$), an average mutational rate per trinucleotide per unit of time $\mu _\text {avg}$, and the activities ${\mathbf {A}}_i (t)$ of each signature such that $\forall t \quad \sum _i {\mathbf {A}}_i (t) = 1$. The $i\text {-th}$ signature is specified by a vector ${\mathbf {P}}_i$ that contains the 96 probabilities of each possible substitution in the trinucleotides context $P^i_{N[K>M]P}$, where $N,P\in \{A,C,G,T\}$, $K \in \{C,T\}$, and $K \ne M \in \{A,C,G,T\}$^1^. The rate of each of the 96 possible substitutions is evaluated as a linear combination between the selected signatures using their activities as weights summed to a background uniform mutational process $P^0$, i.e.,

2 $$\begin{aligned} R_{N[K>M]P} = \mu _\text {avg} \cdot n_{NKP}\left[ (1-\xi ) P^0 + \xi \sum _{i=1}^n { {\mathbf {A}}_i (t) P^i_{N[K>M]P} }\right] , \end{aligned}$$

where $n_{NKP}$ is the number of the trinucleotides with the nucleotide sequence NKP and $\xi$ is a user-defined shrinkage coefficient weighing the signatures against the background (e.g., if $\xi = 1$ all the SNVs will be due to the mutational signatures, if $\xi =0$ all the mutations will be due to the uniform background mutational process). After the generation of the rate matrix, J-SPACE generates the SNVs with the same computational scheme of the previous case (i.e., the Doob-Gillespie algorithm among the branches of the phylogentic tree). Since it was observed that the exposure of the signatures can change during cancer development [28], in J-SPACE it is possible to simulate piece-wise variations of ${\mathbf {A}}_i (t)$. In this case, the user should specify (*i*) a time vector that represents the change points of the signature activities and (*ii*) the values of all the ${\mathbf {A}}_i (t)$ for each time interval.

As a final step, J-SPACE returns the sequences of all samples cells in FASTA format and the related mutation list in textual format.

### Simulating DNA-sequencing

In-silico simulation of NGS data is an expanding field and various simulation tools have been developed [72]. Most tools take as input: (*i*) a genetic sequence (e.g., a reference genome), (*ii*) a set of parameters related to the experimental protocol (e.g., read length) and/or (*iii*) an error model, which may include sequencing errors, PCR artefacts, experimental biases, insertion errors,deletion errors and other [50, 73–77]. In some cases the error models are parameterised empirically from large existing datasets, in other cases they can be generated in a custom way. Importantly, in the former case the error model is platform-dependent, but it allows one to avoid ad hoc arbitrary parameterisations.

For this reason, in order to simulate the reads of a sequencing experiment, J-SPACE relies on the widely-used ART NGS reads simulator [50], which allows one to automatically set the parameters tailored to specific sequencing platforms. More in detail, the user can supply a separate configuration file to specify the error model (for Illumina platforms), the number of reads, the length of the reads, and whether the experiment uses single-end or paired-end/mate-pair reads. In addition, it is possible to insert custom ”calls” to ART in the configuration file. After the execution, J-SPACE returns the simulated reads as FASTQ file for each cell, and the alignment map of the sampled cells’ reads over the genome in SAM and/or ALN format. Note that, in principle, the user can generate the FASTA of the samples without calling ART and could use them as input for other NGS simulation tools that take FASTA files as input.

## Results

We performed different experiments, inspecting different scenarios. We carried out tests to study the cellular dynamics both in 2D and 3D, for different values of driver probability, and for different interaction rules. We analysed the computational time, the influence of spatial constraints on cellular growth and on the molecular evolution of the sequences. Finally, we performed tests to confirm the possibility of using the synthetic NGS reads generated by J-SPACE as input for a single-cell variant calling pipeline. The pipeline and the simulations are available at: https://github.com/BIMIB-DISCo/J-Space.jl/tree/main/Experiments.

### Computational time

To assess the performance of J-SPACE, we measured the computational time necessary to simulate the dynamics and the molecular evolution of many in-silico scenarios.

**Fig. 3:**
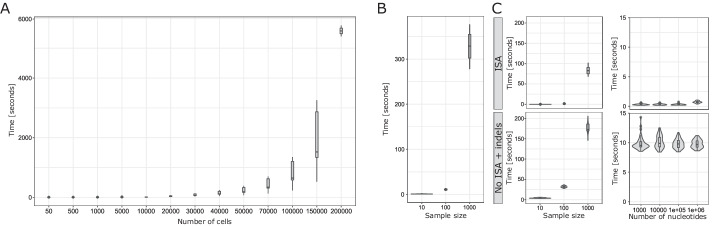
Performances assessment. **A** The distribution of computational time in seconds to perform the simulation described in the text with respect to distinct sample size (over 50 simulation per configuration). **B** The distribution of computational time in seconds to generate the phylogenetic tree with respect to different sample size (over 50 simulation per configuration). **C** Distribution of computational time in seconds to generate the sequences for the phylogenetic trees above, with respect to distinct sample size (left) and genome length (right). In the top row, we present the results of the ISA-based model, in the bottom row we show the results of a finite-sites model (JC69) with indels (see the main text for further details)

First, we run 50 simulations in a 3D regular graph with $10^6$ nodes, with a maximum time of 200 units, a birth rate $=0.4$ per unit of time per cell, death rate $=0.01$ per unit of time per cell, using the contact process and driver probability $\mu _\text {dri} = 0$ per birth event. Results are presented in Fig. 3A. The computational time increases exponentially with respect to the number of simulated cells. However, J-SPACE is able to generate more than $10^{5}$ cells in about one hour.

To evaluate the time necessary to generate the phylogenetic tree from the list of the samples, we simulated the evolution of a single tumour on a 3D regular lattice with 10000 nodes, with maximum time of 300 units, birth rate for unit of time $= 0.4$, death rate for unit of time $= 0.01$, using a contact process, and performed 150 independent samplings, with different sample sizes (10, 100 and 1000 cells, with 50 repetitions each). The distribution of the computational time required to generate the phylogenetic trees related to each sampling is shown in Fig. 3B.

Moreover, for each of the 150 output trees, we evaluated the computational time necessary to simulate the genetic sequences, with distinct genome length ($10^3$, $10^4$, $10^5$ and $10^6$). The evaluation was carried on by comparing the ISA-based simulation versus the case of independent simulation of SNVs and indels^2^.

The results are presented in Fig. 3C (in all cases, the depth of the trees was normalised to 1 [53, 54]). As expected, the infinite-sites model is orders of magnitude faster than the finite-sites model with indels. In addition, the length of the genome leads to a limited increase of the computational load, whereas the computational time increases exponentially with respect to the number of samples. Summarising, we show that J-SPACE is able to simulate long genome sequences ($\approx 10^{6}$ nucleotides) and thousands single cells in a reasonable time. All the computation was performed on a Intel(R) Xeon(R) Gold 6240 @ 2.60GHz.

### Analysis of cancer spatial dynamics and phylogenetic models

We simulated the dynamics of 240 tumours with different driver mutational rates, interaction rules and in both in the 2D and 3D square regular lattice with 5041 and 5832 nodes respectively. The birth rate was set to $\alpha = 0.4$ per unit of time, death rate $\beta = 0.01$ per unit of time, driver mutational probabilities $\mu _\text {dri} =\{0, 10^{-4},10^{-6},10^{-8}\}$ per unit of time, and for a total of 200 units of time.

**Fig. 4:**
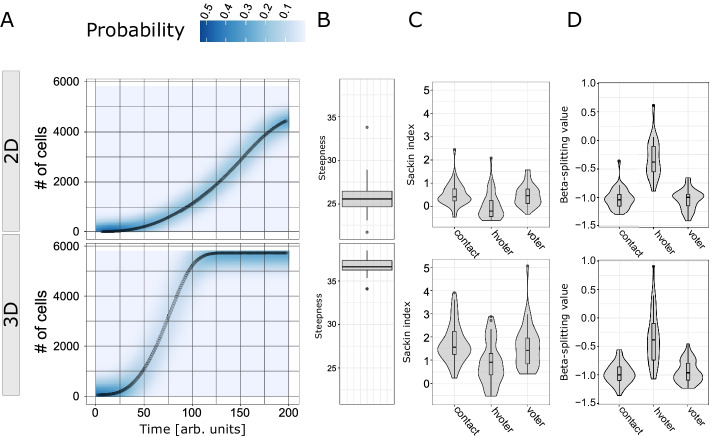
Analysis of cancer spatial dynamics and phylogenetic models. **A** The dynamics of the probability distribution of the number of cells is presented, divided by lattice dimensionality (2D or 3D). The dotted lines represent the expected values.**B** Box plots representing the distribution of the inferred steepness values of logistic growth are presented. **C**–**D** The distribution of the of the Sackin index and Beta-splitting statistic, evaluated on the trees divided by interaction rules and lattice dimensionality

We analysed the dynamics of the number of cells. In Fig. 4A, one can observe the probability distribution and the expected value of the number of cells for different types of lattices. The expected number of cells in a BD process on a lattice follows a logistic growth of the number of cells [63]. We fitted the dynamics of every single run with a logistic curve, and we analysed the distribution of the steepness of such growths Fig. 4B. It is possible to notice that the 3D case has faster growth with respect to the 2D case. This is because in the 3D case, there are more possibilities for cells to replicate. To give a characterisation of the selective pressure between the cells present in the generated tumours and the deviations with respect to a non-spatial simulation, for each of the previous tumours we sampled 100 cells, and we reconstructed their phylogenetic trees. We evaluated the trees balance via the Sackin index (normalised with respect to the pure birth process with no spatial constraints, i.e., the Yule model) [78, 79]. As one can see in Fig. 4C, the distribution of the Sackin index shows that each contact rule has a deviation with respect to the expected Yule model due to the presence of spatial constraints and we notice a strong difference between the 2D and 3D cases. This result is likely due to the fact that the normalised Sackin index considers a star-tree as more balanced with respect to with a fully symmetric tree [80]. For instance, the 2D case has a higher genetic drift due to the spatial constrains and exhibits a star-like structure.

We also measured the Beta-split statistic [79, 81], which evaluates the diversification rates between cells, and the results are presented in Fig. 4D. We observe that the hierarchical voter model shows a more substantial diversification rate, due to the strong advantage of bearing driver mutations.

### Analysis of synthetic sequencing data

**Fig. 5:**
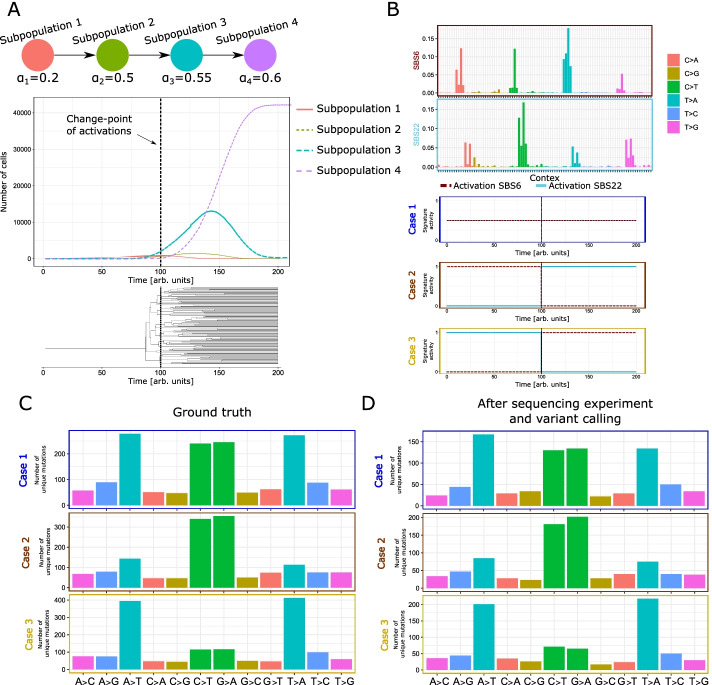
Variant calling with different mutational signatures. **A** An example dynamics of the number of cells for each subpopulation generated during the simulation. The bottom part of the panel presents the input driver mutational tree with the birth rate for each subpopulation. **B** At the top we present the phylogenetic tree generated by sampling 100 cells. We proceeded by simulating three different substitution models generated combinations of signatures SBS6 and SBS22 from the COSMIC database [71]. The difference between the three models consists in the time dynamics of the activation functions presented in this figure. **C** The count of the number of unique mutations simulated divided per class of substitution. The plot presents the result for the three different models. **D** The count of the number of unique mutations divided per class of substitution detected using the pipeline described in the main text

We simulated a single tumour using an hierarchical voter process in a 3D square regular lattice with 42875 nodes. The death rate was set to $\beta = 0.01$ per unit of time, the driver mutational probability to $\mu _\text {dri} = 0.01$ per unit of time, for a total of 200 units of time. In this case, we fixed a linear mutational tree with 4 driver mutations. The birth rate of each subpopulation and the mutational tree are presented in Fig. 5. In the same figure, we also show the cell population dynamics. It is possible to notice that the last subpopulation performs a clonal sweep in the latest part of the simulation. From this tumour, we sampled 100 cells and we present the related phylogenetic tree (Fig. 5B). The tree has a very long initial branch ($\approx 82$ time units), due to the fact that we sampled only cells of the subpopulation 4 and that the least common ancestor of such cells is the first cell bearing the corresponding driver mutations. For this tree, we generate the sequences of the samples with three different substitution models, composed by distinct linear combinations of signatures SBS6 (a mutational process associated with defective DNA mismatch repair) and SBS22 (associated to the exposure to aristolochic acid) with different activation functions. In detail, we imposed: *i)* a constant activity for both signatures with values of $A_{SBS6}(t)=0.5$ and $A_{SBS22}(t)=0.5$, *ii)* the presence of a change-point of the activities at 100 units of time, i.e., in the first time span only SB22 is active $A_{SBS6}(t<100)=0$ and $A_{SBS22}(t<100)=1$, in the second time span the activations are exchanged, i.e., $A_{SBS6}(t \ge 100)=1$ and $A_{SBS22}(t\ge 100) = 0$, *iii)* an opposed time-dependent activation pattern with respect to the previous one (see Fig. 5B). The other parameters of the simulation are the following: an ancestral genome with 10000 bases with following composition $\nu _A= 0.3$, $\nu _C= 0.2$, $\nu _G= 0.2$, $\nu _T= 0.3$, the average mutational rate $\mu _{\text {avg}} = 10^{-3}$ per trinucleotide and unit of time, the ratio between signature mutation and background of $\xi = 0.8$.

In Fig. 5C, we present the counts of the number of unique mutations divided per class of substitution. In this plot it is evident the effect of the change-point in the activities of the signatures. In particular, due to the structure of the phylogenetic tree (with a very long initial branch), the number of unique SNVs related to the signature that is activated at $t < 100$ is smaller with respect to the other signature. This behaviour is expected and shows that, with J-SPACE, it is possible to study the combination of spatial dynamics, clonal evolution and time-dependent substitution models.

Finally, for all the samples of the previous examples, we simulated an Illumina HiSeq 2500 paired-end sequencing experiment, with 100 average reads per cell, mean read length $= 100$ bases, and DNA fragment size of $= 200 \pm 10$ bases.

To analyse the FASTQ files so generated, we used the following bioinformatics pipeline. First, we created the indexing and dictionary of the reference FASTA.

Second, the paired-end reads (FASTQ) were aligned using BWA-mem2 [6], and duplicate reads of a sequence fragment originated from PCR duplication artefacts were removed.

Third, SNVs and indels calling was performed followed by a standard set of filtering steps.

Fourth, we retrieved the count of the number of unique mutation simulated divided per class of substitution from the VCF files. The plot is presented in Fig. 5D. We see how with this experiment we detect a smaller number of mutations, either due to the poor quality or the low coverage. However, in this experimental scenario it is possible to observe the same effect described in the GT case (see Fig. 5C). The complete simulation, the variant calling pipeline, the FASTQ, the BAM/SAM, the GT sequences of the samples, the phylogenetic tree, and the VCF files can be downloaded at: https://github.com/BIMIB-DISCo/J-Space.jl/tree/main/Experiments/Experiment_Pipeline.

## Conclusion

We introduced J-SPACE, a framework to simulate the spatial dynamics of a multi-cellular system and, especially, of tumour subpopulations. J-SPACE is specifically designed to efficiently simulate the heterogeneous behaviour of the spatial growth of cancer cells and returns a rich output, which is useful to analyse the emergent dynamics, the consequences of incomplete spatial sampling and those of experiment-specific errors. We tested the outputs in various in-silico scenarios to test if J-SPACE replicates the influence of spatial constraints on cellular growth and on the generated phylogenetic trees. Finally, we showed how is possible to use the synthetic NGS reads generated by J-SPACE as input for a single-cell variant calling pipeline. Accordingly, J-SPACE can be used to produce synthetic datasets to test bioinformatics tools that process either bulk or single-cell cancer sequencing data. J-SPACE is distributed as a Julia package freely available to the community.

Several improvements of J-SPACE are underway, with the main objective of delivering a more biologically faithful representation of cancer evolution, including (but not limited to): (*i*) the design of evolutionary models of large structural variations, such as copy-number alterations and gene fusions, (*ii*) the definition of an explicit model of cell differentiation/specialisation, (*iii*) the simulation of the interaction between different cell types, including stroma and extra-cellular matrix, (*iv*) the modelling of external interventions, such as pharmacological treatments or therapeutic strategies.

## Data Availability

The datasets generated and/or analysed during the current study are available in the github repository: https://github.com/BIMIB-DISCo/J-Space.jl.

## Abbreviations

BD: Birth-death
NGS: Next-generation sequencing
SNVs: Single nucleotide variants
ISA: Infinite allele assumption
OGA: Optimized Gillespie algorithm
JC69: Jukes and Cantor 1969
F81: Felsenstein 1981
K80: Kimura 1980
HKY85: Hasegawa et al. 1985
TN93: Tamura and Nei 1993
K81: Kimura 1981
GT: Ground truth
VAF: Variant Allele frequency
SBS: Single base substitution

## Footnotes

1 There are 96 possible substitutions because, in the signature discovery process, only the pyrimidines are considered. Accordingly, there are only six different possible substitutions C>A, C>G, C>T, T>A, T>C, and T>G and, if we consider two flanking bases, we have 96 classes of substitution.

2 Mutational rate of $\mu _\text {neut} = 10^{-6}$ for the ISA-based case; JC69 model with $\mu = 10^{-6}$ per unit of time, with maximum indel length of $L_\text {indel} = 100$ bases, and $\mu _{\text {indel}} = 10^{-8}$ per unit of time, and Lavalette parameter $a=0.5$, for the latter scenario.
